# Intercurrent and opportunistic infections in patients treated with biological agents: study hypothesis

**DOI:** 10.1186/1471-2334-14-S7-P40

**Published:** 2014-10-15

**Authors:** Ramona Ștefania Popescu, Andra Bălănescu, Adrian Streinu-Cercel

**Affiliations:** 1 Carol Davila University of Medicine and Pharmacy, Bucharest, Romania; 2 National Institute for Infectious Diseases "Prof. Dr. Matei Balş", Bucharest, Romania; 3 “Sf. Maria” Clinical Hospital, Bucharest, Romania

## Background

Biologic therapy has been used for over 10 years and is increasingly used in the treatment of many chronic inflammatory diseases. At the present time it has a very broad range of applications: rheumatic, dermatologic, gastro-intestinal, neurologic and neoplastic diseases. Biologic therapy is represented by immunomodulator agents (antibodies or other peptides) that interfere with the regular humoral immune response. Thus, in addition to the beneficial effects in relation with the underlying disease it may lead to opportunistic infections: bacterial, viral, fungal, or parasitic.

## Study hypothesis

Opportunistic infections in patients on biological therapy may represent cause for concern, given the increased risks of infection in this category of patients. Our objective is to conduct a study on two research directions:

• 1) A prospective, observational study which will consist of a group of patients undergoing treatment with biologic agents and a control group. Study visits will occur at the baseline, and then every 24 weeks plus unscheduled visits when required in case of infections. We will evaluate the following parameters: blood tests (CBC, biological inflammatory syndrome, coagulation, biochemistry), cytokines values, QuantiFERON TB Gold, urine analysis, blood cultures and nasal, pharyngeal and axillary carriage.

• 2) A retrospective study (statistical analysis) of the already existing data collected in Romanian Registry of Rheumatic Diseases which contains an up to date list of all the patients with RA (within the country) currently undergoing biological therapy.

## Expected Results

The objectives of the study are: to determine the rate of occurrence of intercurrent and opportunistic infections in patients undergoing biological therapy within the country (data which is not available at the moment), to compare this information with the available external data and to define a particular profile for patients susceptible to secondary infections due to biological therapy.

